# Myosin-19 and Miro Regulate Mitochondria–Endoplasmic Reticulum Contacts and Mitochondria Inner Membrane Architecture

**DOI:** 10.3390/cells14211657

**Published:** 2025-10-23

**Authors:** Aya Attia, Katarzyna Majstrowicz, Samruddhi Shembekar, Ulrike Honnert, Petra Nikolaus, Birgit Lohmann, Martin Bähler

**Affiliations:** 1 Institute of Integrative Cell Biology and Physiology, University of Münster, Schlossplatz 5, 48149 Munster, Germany; 2 Medical Molecular Genetics Department, National Research Centre, Dokki, Cairo 12311, Egypt

**Keywords:** mitochondria, Myosin 19, outer mitochondrial membrane, Miro1/2, TRAK, cristae, OXPHOS

## Abstract

Mitochondrial dynamics are important for cellular health and include morphology, fusion, fission, vesicle formation, transport and contact formation with other organelles. Myosin XIX (Myo19) is an actin-based motor, which competes with TRAK1/2 adaptors of microtubule-based motors for binding to the outer mitochondrial membrane receptors Mitochondrial Rho GTPases 1/2 (Miro). Currently, it is poorly understood how Myo19 contributes to mitochondrial dynamics. Here, we report on a Myo19-deficient mouse model and the ultrastructure of the mitochondria from cells of Myo19-deficient mice and HEK cells, Miro-deficient HEK cells and TRAK1-deficient HAP1 cells. Myo19-deficient mitochondria in MEFs and HEK cells have morphological alterations in the inner mitochondrial membrane with reduced numbers of malformed cristae. In addition, mitochondria in Myo19-deficient cells showed fewer ER–mitochondria contact sites (ERMCSs). In accordance with the ultrastructural observations, Myo19-deficient MEFs had lower oxygen consumption rates and a reduced abundance of OXPHOS supercomplexes. The simultaneous loss of Miro1 and Miro 2 led to a comparable mitochondria phenotype and reduced ERMCSs as observed upon the loss of Myo19. However, the loss of TRAK1 caused only a reduction in the number of cristae, but not ERMCSs. These results demonstrate that both actin- and microtubule-based motors regulate cristae formation, but only Myo19 and its membrane receptor Miro regulate ERMCSs.

## 1. Introduction

Mitochondria are intracellular organelles known as the powerhouses of the cell. They provide the cell with the required energy (ATP) through the OXPHOS process. But in addition, they regulate other cellular functions such as calcium homeostasis, metabolism and apoptosis. Mitochondria have a double membrane, namely an outer and an inner membrane (OMM and IMM, respectively). The IMM is densely folded up into cristae [1,2]. Embedded in the cristae membrane is the electron transport chain and ATP synthase. To maintain the cristae structure, a mitochondrial contact site and cristae organizing system (MICOS) is needed [3]. It consists of a well conserved multi-subunit protein complex (~>1 MDa) that is inserted in the inner membrane. The MICOS complex, together with the sorting and assembly machinery (SAM) complex, forms the mitochondria intermembrane bridging complex (MIB) that connects the IMM to the OMM [4]. Depletion of MICOS components leads to altered cristae morphology and defective oxidative phosphorylation [5]. In addition to the MICOS, the cristae structure is further controlled by dimer formation of the mitochondrial ATP synthase, inner membrane remodeling by a dynamin-related GTPase (Mgm1/OPA1), and modulation of the mitochondrial lipid composition [6].

Mitochondria are very dynamic and active eukaryotic organelles. They undergo a series of continuous events of fusion and fission, form contacts with other membranous organelles such as endoplasmic reticulum (ER) and peroxisomes and move along cytoskeletal tracks to sites where they are needed [7]. The interplay between mitochondria and other organelles is crucial for the maintenance of their function, exchange of metabolites, cell survival regulation and intracellular signaling [8]. The best studied membrane contact sites are those formed with the endoplasmic reticulum (ER). They are called the ER–mitochondrion contact sites (ERMCSs) in mammals. ERMCSs play a crucial role in phospholipid synthesis and transfer, calcium signaling (Ca^2+^), fission and fusion, regulation of oxidative stress and inflammatory responses, bioenergetics and cell survival [9,10,11,12,13]. ERMCSs require proteins on both the ER and the mitochondria to bridge the two organelles. Regulated actin polymerization at the contact sites can initiate mitochondrial fission [14].

Motor proteins were shown to bind mitochondria and exert force along microtubules and actin filaments and to contribute to mitochondrial dynamics [15,16,17]. Mitochondria Rho GTPases (Miro1/2) inserted in the OMM are receptors for the motor proteins. They bind through adaptors TRAK1/2 to the microtubule-based motors dynein and kinesin [18,19,20] and directly to the actin-based motor Myo19 [21]. Myo19 is stabilized by Miro1/2 and is degraded upon the loss of Miro1/2 [21,22]. By contrast, Miro1/2 and TRAK1 levels were not affected by the loss of Myo19 [17,21]. Myo19-deficient cells exhibit a decreased OXPHOS rate, increased ROS production, asymmetric partitioning of mitochondria to daughter cells and stochastic failure of cytokinesis [17,23,24]. More recently, it was shown that Myo19 and Miro proteins are required for normal cristae architecture and for proper ERMCS formation [24,25,26]. Myo19 and Miro proteins were found to be linked to components of the MICOS. It was proposed that the linkage of Myo19 and Miro to the MICOS allows for mechanical coupling of the OMM and IMM and thereby regulates cristae architecture [24,25].

To further investigate these observations, we asked whether Myo19 is required for mitochondrial ultrastructure and ER–mitochondria contact sites in vivo. To this end, we generated and characterized Myo19-deficient mice. Myo19-deficient mice did not show any obvious phenotype under standard housing conditions. However, ultrastructural analysis confirmed that the loss of Myo19 induced alterations in mitochondrial morphologies and ERMCS abundance in cells. To determine to what extent Myo19, its receptor Miro1/2 and the adaptor TRAK1 are components of the same pathway, the ultrastructure of the mitochondria was directly compared in Myo19-deficient, Miro1/2-double-deficient and TRAK1-deficient cells. These results suggest that actin- and microtubule-based force generation differently affect the ultrastructure of mitochondria and ERMCSs.

## 2. Material and Methods

### 2.1. Mice

All mice were housed with the ethical permit, in the animal facility of the university of Münster, Institut für Molekulare Zellbiologie, according to the national and European legislation. Animal experiments were carried out according to European Community guidelines (86/609/EEC, 2010/63/EU). C57Bl/6 wild-type (WT) mice were obtained from Harlan Winkelmann Laboratories (Borchen, Germany). Myo19^tm1a(EUCOMM)Hmgu^ “knockout first allele” heterozygous mice were created by the mouse production service of the EC FP7 funded Infrafrontier-I3 project (Lluis Montoliu, CNB-CSIC, Madrid, Spain) using the ES Cell Clone HEPD0543_9_A05 produced by EUCOMM/Hmgu. Mice were backcrossed onto C57Bl/6.

### 2.2. Genotyping of Mice

Genotyping of mice was performed by multiplex PCR with specific forward MB1178F (GCTTGGCATGGAGTTGTAAGCC) and reverse MB1179R (CACAACGGGTTCTTCTGTTAGTCC), MB1180R (GCATAGAACTTCATCAGGCATCG) primers.

The PCR reaction was prepared on ice and included PCR buffer (Taq buffer 10×), 2.5 mM each of dNTP (dATP, dCTP, dGTP and dTTP), 10 pmol/µL of forward primer and 5 pmol/µL of each reverse primer, 1 µL of Taq polymerase and 1.5 µL of DNA template. The PCR reaction was performed as a touchdown hot-start PCR in a preheated thermocycler.

Amplified fragments were separated in a 1% agarose gel with 0.5 µg/mL of ethidium bromide in 1× TAE buffer at 70 V. Bands were visualized using the BioDoc Analyze gel documentation system (Biometra, Jena, Germany). Genotyping of mice by multiplex PCR yielded a fragment length of 584 bp for the wild-type allele and of 289 bp for the mutant allele.

### 2.3. Antibodies and Stains

The following monoclonal and polyclonal antibodies were used: anti-β-actin (WB: 1 µg/mL, AC-15/A1978, Sigma-Aldrich, St. Louis, MO, USA), anti-Miro1/2 (WB: 0.25 µg/mL, NBP1-59021, Novus Biologicals, Centennial, CO, USA), anti-MTCO1 (BN-PAGE, 1:1000, ab14705, Abcam, Cambridge, UK), anti-UQCRC2 (BN-PAGE 1:1000, ab14745, Abcam). anti-human Myo19 (WB: 0.176 µg/mL, ab174286, Abcam), anti-mouse Myo19 (WB: 1 µg/mL, sc-248029, Santa Cruz, Dallas, TX, USA), anti-paxillin (WB: 2 µg/mL, MA5-13356, Invitrogen, Waltham, MA, USA), anti-Phospho-Paxillin (PY118, WB: 1 µg/mL, 44-722G, Thermo Fisher Scientific, Waltham, MA, USA), anti-VDAC1 (WB: 1 µg/mL, ab14734, Abcam), anti-vinculin (WB: dilution 1:1000, V9131, Sigma-Aldrich), anti-mouse IgG-HRP (Goat, WB: dilution 1:5000, 115-035-003, Jackson ImmunoResearch, West Grove, PA, USA), anti-rabbit IgG-HRP (goat, WB: dilution 1:5000, 111-035-003, Jackson ImmunoResearch) and anti-mouse-IgG-Alexa Fluor 488 (goat, IF: 1:500, 115-545-003, Jackson ImmunoResearch).

Mitochondria were stained with Mitotracker Orange CMXRos (50 nM, M7510, Thermo Fisher Scientific) and F-actin with FITC–phalloidin (IF: dilution 1:100, P5182, Sigma-Aldrich).

### 2.4. Cell Culture

#### 2.4.1. Derivation, Immortalization and Culture of Mouse Embryonic Fibroblasts (MEFs)

A female mouse at day 12.5 of pregnancy was sacrificed by cervical dislocation and the uterine horns were removed and placed in ice-cold 1× PBS. Embryos were separated from the placenta and embryonic sac and immersed in 1× PBS. After dissection of the head and red organs (heart, liver and extremities), the remaining embryos were finely minced using a sterile razor blade and digested for 4 min at 37 °C with 0.05% Trypsin-EDTA. Afterwards, 50 mL of freshly prepared MEF medium [DMEM including NaHCO_3_ 3.7 g/L, L-glutamine 0.58 g/L, D-glucose 4.5 g/L, Phenol red, supplemented with penicillin 100 U/mL, streptomycin 100 µg/mL (Pan Biotech, Aidenbach, Germany), L-glutamine 0.29 g/L, β-mercaptoethanol 10 µM and heat inactivated FCS 10% (*v*/*v*) (Biochrom, Cambridge, UK) was added to inactivate the trypsin and dilute the DNA. Cells were further dissociated by thoroughly pipetting up and down and filtered through a 100 µm cell strainer. The cell suspension was centrifuged at 200× *g* for 5 min, and the cell pellet was resuspended in 10 mL of MEF medium. Cells were plated onto gelatinized 10 cm plates (10 mL of 0.1% gelatin for 30 min at 37 °C) at a density of one embryo per plate and incubated in a humidified incubator at 37 °C in 5% CO_2_ and 95% humidity. For passaging, a 70–80% confluent cell layer was washed with 1 × PBS and digested with 0.05% Trypsin-EDTA for 2–5 min at 37 °C. Next, 10 mL of fresh medium was added to stop the reaction, and cells were centrifuged at 300× *g* for 5 min. Resuspended cells were expanded at a dilution of 1:3 every other day, when cell layers reached sub-confluency.

To immortalize primary MEFs of each genotype during the 4th passage, they were seeded in a 6-well dish at two dilutions: 1:4 and 1:6 and grown overnight at 37 °C. On the next day, cells were transfected with 2 µg SV40 T-antigen plasmid (Simian virus-40 large-T antigen) [27] using Lipofectamine^®^ LTX Plus Reagent (Thermo Fisher Scientific, Waltham, MA, USA) for 4 h, washed twice with fresh MEF medium and incubated overnight at 37 °C. Two days after transfection, confluent cells were split into 10 cm gelatinized plates (P1). To select positive clones, cells were split at a high (1:4) and low density (1:10) every 3–4 days until the sixth passage (P6, 1:100,000-fold splitting of the original cells). Once the cultures presented a homogeneous cell population and started to grow steadily, MEFs were considered as immortalized and used further for experiments.

#### 2.4.2. Generation of Genetically Modified Knockout Cell Lines:

The HEK Myo19 knockout cell line was generated by CRISPR/Cas9-mediated genome editing as previously described [17]. Cells were maintained in complete Dulbecco’s Modified Eagle Medium (DMEM medium supplemented with 10% Fetal Calf Serum (FCS), 100 U/mL of Penicillin and 100 µg/mL of Streptomycin).

To generate Miro DKO cells, HEK 293T WT cells were transfected with pX330-Miro1ex7 (gift from Kornmann lab [28]). The resulting clones after FACS sorting of transfected cells were grown and subsequently checked for Miro1KO by PCR sequencing and Western blotting. For knockout of Miro2, CRISPR plasmid was obtained from Santa Cruz Biotechnology (Dallas, TX, USA, sc-431979). Miro1 KO cells were transfected with this plasmid and the same procedure of obtaining and screening the clones was followed to obtain Miro DKO cells.

HAP1 TRAK1 knockout cells (HZGHC006545c001) were obtained from Horizon Discovery (Horizon Genomics GmbH, Cambridge, UK). Cells were edited by CRISPR/Cas to contain an 11bp deletion in exon 7 of TRAK1. Cells were cultured in complete Iscove’s Modified Dulbecco’s Medium (IMDM) (IMDM media supplemented with 10% FCS, 100 U/mL of Penicillin and 100 µg/mL of Streptomycin).

#### 2.4.3. Preparation of Cell and Tissue Homogenates

HEK cells were directly detached in medium and washed twice with ice-cold 1× PBS by centrifugation (300× *g*, 5 min at 4 °C). The cell pellet was resuspended in an appropriate amount of ice-cold NP-40 lysis buffer [50 mM TrisHCl, pH 7.4, 10% (*v*/*v*) Glycerol; 100 mM NaCl, 2 mM MgCl2, 1% (*v*/*v*) NP40, freshly added: 1 mM DTT, 10 µg/mL of aprotinin, 10 µg/mL of leupeptin, 10 µg/mL of Pefabloc]. Protein concentration was determined by Bradford Assay using BSA as a standard. Cell homogenates were mixed with 5× Laemmli sample buffer (Tris/HCl (pH 6.8) 0.1 M, EDTA 5 mM, SDS 15% (*w*/*v*), sucrose 40% (*w*/*v*), β-mercaptoethanol 10% (*v*/*v*), Bromophenol Blue 0.02%), to a final concentration of 1× sample buffer and boiled at 100 °C for 5 min. Prepared samples were stored at −20 °C.

Organs were collected from mice, washed with ice-cold 1× PBS and directly frozen in liquid nitrogen. The weight of each organ was determined and a 10-fold volume (*v*/*w*) of ice-cold tissue homogenization buffer (HEPES (pH 7.4) 15 mM, sucrose 320 mM with freshly added DTT 1 mM, Leupeptin 10 µg/mL, Aprotinin 10 µg/mL and Pefabloc 10 µg/mL) was added. Tissues were mechanically homogenized with a Potter–Elvehjem tissue homogenizer at 4 °C. Protein concentrations were determined by Bradford Assay. Samples were mixed with 5× Laemmli sample buffer, boiled at 100 °C for 5 min and stored at −80 °C.

### 2.5. Western Blotting

Cell and tissue homogenates were separated by sodium dodecyl sulfate polyacrylamide gel electrophoresis (SDS-PAGE). Proteins were electrophoretically transferred overnight on to PVDF membrane (03010040001, Roche, Sigma-Aldrich). The membrane was blocked in 5% non-fat dry milk in TBS with 0.05% Tween 20 (TBST) for 1 h at room temperature (RT) and incubated with primary antibodies overnight at 4 °C. The membrane was washed three times with TBST (each wash 5 min) and incubated with the corresponding HRP-conjugated secondary antibody for 1 h at RT. After three washes with TBST, the protein bands were detected with Super Signal West Pico Substrate (34078, Thermo Fisher Scientific) according to the manufacturer’s protocol, using a ChemiDoc MP Imaging System (Bio-Rad, Hercules, CA, USA).

### 2.6. Histology

#### 2.6.1. Hematoxylin and Eosin (H&E) Staining

Tissues were embedded in paraffin as described [29]. Paraffin embedded sections were deparaffinized and rehydrated by passaging them twice through Roticlear for 10 min followed by a graded ethanol series: twice 100% ethanol for 10 min, once 96% ethanol for 5 min and 70% ethanol for 5 min. Slides were then washed twice with ddH_2_O for 5 min each and incubated in Mayers hemalum solution (#T865.3, Roth, Karlsruhe, Germany) for 4 min. To remove excess stain, slides were washed several times with ddH_2_O for 10 min each. Eosin staining was performed by incubation of slides with 0.5% eosin G-solution (#X883.1, Roth) for 1 min and subsequent washing in ddH_2_O for 10 min. Sections were then dehydrated again by washing in 70% and 96% ethanol for 30 s each and two times in 100% ethanol for 5 min each. Next, slides were submerged in Roticlear twice for 5 min each and mounted with Roti-Mount medium (#HP68.1, Roth) and coverslips. Mounted slides were dried overnight at RT.

#### 2.6.2. Preparation of Tissues and Cultured Cells for Transmission Electron Microscopy

Cells (MEFs, HEK and Hap1 cells) were seeded on coverslips in 24-well plates for 24–48 h before being fixed. Half of the DMEM cell medium was replaced by 4% paraformaldehyde and 4% glutaraldehyde in cacodylate buffer (100 mM sodium cacodylate pH 7.4, 2 mM CaCl_2_) and incubated for 10 min. The mixture of medium and buffer was removed and replaced by 2% paraformaldehyde and 2% glutaraldehyde in cacodylate buffer for 2 h at RT. Cells were washed three times with cacodylate buffer and post-fixed with 1% osmium tetroxide in cacodylate buffer for 30 min on ice followed by 30 min at RT. Afterwards samples were washed in distilled water. They were dehydrated in a graded ethanol series starting with 70% ethanol overnight at 4 °C followed by two times 90% ethanol and 96% ethanol each for 15 min. at RT. Finally, samples were incubated 3 × 30 min. at room temperature with 100% anhydrous ethanol. Samples were infiltrated with increasing concentrations of embedding medium freshly prepared by mixing 60% (*v*/*v*) Epon I (37.48 g Epon 812 (Serva, Heidelberg, Germany), 49.50 g dodecenyl succine anhydride (Agar, Rotherham, UK) and 40% (*v*/*v*) Epon II (60.45 g Epon 812, 52.98 g methylnadic anhydride (Serva). Samples were incubated at RT for 1 h in 3:1 anhydrous ethanol and embedding medium, followed by 1:1 and 1:3 anhydrous ethanol and embedding medium, respectively. Afterwards samples were incubated under the hood in 100% embedding medium overnight at RT followed by replacement with embedding medium. Gelatine capsules with removed bottom were placed on the coverslips with attached cells and a thin layer of embedding medium and surface-dried for ~6 h at 60 °C. Subsequently, the capsule was completely filled with embedding medium and polymerized at 60 °C for 72 h. The coverslip was carefully removed by alternate incubation of the polymerized resin in liquid nitrogen and hot water. The Epon blocks were trimmed, and ultrathin (50 nm) sections were cut using an ultramicrotome (Ultracut E, Reichert-Jung, Leica Microsystems GmbH, Wetzlar, Germany). Sections were mounted on 75 mesh fomvar-coated copper grids (PLANO) and contrasted with 1% uranyl acetate for 30 min.

### 2.7. Image Acquisition and Analysis

Fluorescence images were acquired using a 63×/1.4 NA objective mounted on a spinning disk confocal laser-scanning microscope (UltraVIEW VoX, PerkinElmer; Eclipse Ti, Nikon, Tokyo, Japan). Z-stacks across the depth of the cell were acquired for each experiment and total projections were calculated.

Ultrastructural images were acquired using a Zeiss Libra120 transmission electron microscope (TEM) (Zeiss, Oberkochen, Germany). For each experimental condition, multiple sections were screened, and one representative section was selected for imaging. Panoramic views of entire cells were acquired, capturing all mitochondria within the cell. Typically, images of 5–6 cells were taken per experimental condition, and an average of 2 fully visible cells per condition were selected for quantitative analysis. The number of mitochondria per cell varied in a section. A total of 6–8 cells were analyzed in detail for each cell type.

Mitochondrial morphology (area and length), cristae count and number as well as the ER–mitochondria contacts were assessed by analyzing TEM images using ImageJ [30]. Mitochondrial area and perimeter were determined by outlining the mitochondria using the region of interest (ROI) tool in ImageJ. Cristae analysis and scoring were performed by counting the total number of cristae per mitochondrion and tracing the outline of each crista within the mitochondrion. The ER–Mitochondria contacts were limited to 35 nm distance. No mitochondria were excluded from the analysis unless their structures were not clearly visible.

### 2.8. Oxygen Consumption Rate Measurements

The oxygen consumption rate (OCR) was measured by an automatic flux analyzer XF 96 (Seahorse/Agilent, Santa Clara, CA, USA) as described in Arroum et al. (2023) [31]. Briefly, one day before the experiment 30,000 MEFs or HAP1 cells were plated in complete DMEM (MEFs) or IMDM (HAP1) in a 96-well XF Cell Culture Microplate. The sensor cartridge was calibrated with 200 µL of sterile water per well and incubated at 37 °C, environmental CO_2_. The following day, water in the sensor cartridge was removed and replaced by 200 μL of XF Calibrant per well and incubated for 60 min at 37 °C and environmental CO_2_. Just before starting the measurements, the cells were incubated in 180 μL XF Base Assay Medium (25 mM D-glucose, 1 mM pyruvate and 2 mM L-glutamine in XF Base Medium) for 1 h at 37 °C and environmental CO_2_. After calibration with the Hydrate Sensor Cartridge, the Seahorse XF 96 Cell Culture Microplate (Agilent Technologies, Chicopee, MA, USA) was mounted in the XF 96 Analyzer. First, basal respiration was determined. Then, through sequential inhibitor injections of (1) oligomycin [2 µM]; (2) carbonylcyanid-p-trifluoromethoxyphenylhydrazon [FCCP] [0.5 µM]; and (3) rotenone [0.5 µM]), the ATP synthesis-related respiration, the maximum respiration, the proton leak and the non-mitochondrial respiration were determined at 37 °C, respectively. After finishing the measurements, the cells were stained with Hoechst 33,342 and counted with the Cytation 1 Cell Imager (Agilent) for normalization to cell number.

### 2.9. Blue Native Gels

To perform BN-PAGE, cells were harvested by scraping from two confluent T175 flasks for each of WT and KO MEFs and processed as described in [32]. Harvested MEFs were mechanically lysed by using a cell homogenizer. Then mitochondria were enriched via differential centrifugation. Digitonin (20% [*w*/*v*] stock solution, TLC grade; Sigma-Aldrich, #D5628) was used for mitochondrial solubilization at a digitonin-to-protein ratio of 6:1 (*w*/*w*), as described in [33]. In total, 100 μg of protein were loaded per lane and separated via a vertical 3–12% native SERVAGel™ (#43251.01, Serva, Heidelberg, Germany). NativeMark™ (Thermo Fisher, #LC0725) unstained was used as a protein standard for molecular weight estimation. All buffers were prepared as mentioned in Wittig et al. (2006) [34]. Separated protein complexes were electrophoretically transferred to PVDF membrane for immunoblotting.

### 2.10. Statistical Analysis

Data were analyzed using OriginPro 2015 SR2 (OriginLab Corporation, Northampton, MA, USA) and GraphPad Prism 10.3.1 (GraphPad Software, San Diego, CA, USA). For pairwise comparisons, Mann–Whitney U-tests were used, while for multiple comparisons, one-way ANOVA was performed with a post hoc Bonferroni test. Data are presented showing mean ± SEM or ± SD as indicated. Significance values are indicated as * *p* ≤ 0.05; ** *p* ≤ 0.01; *** *p* ≤ 0.001 and **** *p* ≤ 0.0001.

## 3. Results

### 3.1. Characterization of Myo19 Knockout Mouse Model

**Figure 1:**
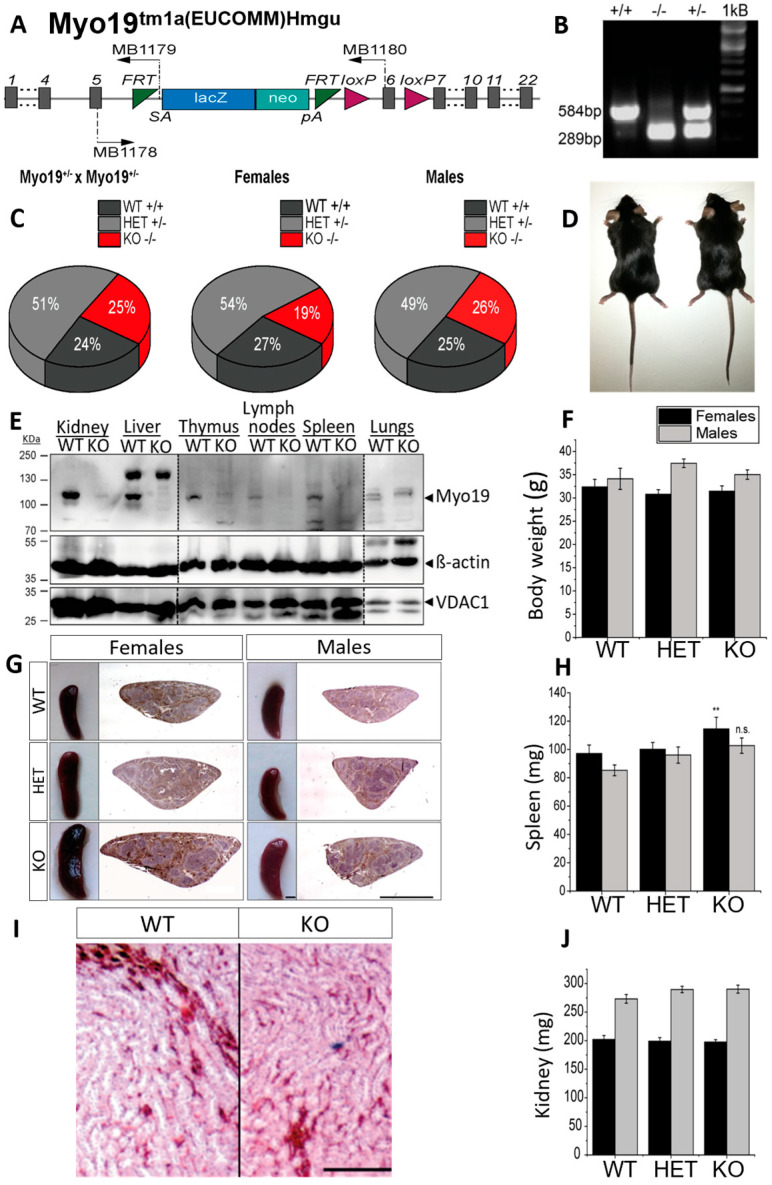
Characterization of Myo19 transgenic mice. (**A**) Allele map of the recombinant Myo19 gene locus with an inserted reporter cassette. Gray bars represent exons; SA—En2 splice acceptor; pA—SV40 polyadenylation site. MB1178-80 denote the primers used for genotyping. (**B**) Genotyping of mice yielded a fragment of 584 bp for the wild-type allele (+) and of 289 bp for the recombined Myo19 allele (−). Both fragments are detected in heterozygous mice (HET, +/−). (**C**) Heritability of genome-edited Myo19 alleles. Breeding of heterozygous mice yielded offspring at the Mendelian ratios. Gender frequencies from HET × HET matings are indicated. N = 71. (**D**) Representative adult wild-type (*+/+*, left) and Myo19 knockout (*−/−*, right) mice are shown. (**E**) Tissue expression of Myo19 protein. Immunoblot analysis of tissue homogenates from WT and Myo19 KO mice were probed with the indicated antibodies. Tissues are indicated at the top of the panels. Several separate blots were assembled as marked by dashed lines. (**F**) Determination of body weight of adult male and female mice of the indicated genotypes. (**G**) Myo19 knockout female mice exhibit splenomegaly. Images of mouse spleens and corresponding histological sections of the indicated genotype and gender are shown. Sections were stained with Hematoxylin and Eosin (H&E). Scale bar, 0.2 cm. (**H**) The weight of isolated spleens of adult female and male mice of the indicated genotypes was determined (n ≥ 6). Error bars represent ± SEM. Statistical significance for multiple group comparisons was determined by one-way ANOVA with Bonferroni post hoc test. Spleen weight differences: females, *p* = 0.005; males, *p* = 0.052 ** *p* ≤ 0.01; n.s. not significant. (**I**) Histology of kidney sections (H&E staining) from wild-type and knockout mice. Shown are regions from the renal medulla with renal tubuli and associated blood vessels. Scale bar: 50 µm. (**J**) Kidney weight measurements (mean ± SEM) from adult female and male mice of the indicated genotypes.

To examine Myo19 function in vivo, we generated Myo19^−/−^ knockout (KO) mice by homologous recombination (Figure 1A,B). Myo19^−/−^ mice were obtained by crossing heterozygotes (Myo19^+/−^ × Myo19^+/−^). The offspring genotype distribution matched the expected Mendelian ratio (Figure 1C). Myo19^−/−^ females were slightly underrepresented, whereas Myo19^−/−^ males were born at the expected ratio (Figure 1C). Matings of Myo19^−/−^ mice (KO × KO) gave rise to progeny suggesting that Myo19^−/−^ mice are fertile. Sperm motility, testis weight, and histology were comparable between KO and control males (Supplementary Figure S1A,B). Myo19^−/−^ mice showed no obvious phenotype (Figure 1D). The body weight of Myo19^−/−^ mice was comparable to the body weight of wild-type mice (Figure 1F).

Next, tissue homogenates from wild-type and globally Myo19^−/−^ mice were prepared, and the Myo19 protein expression analyzed. Myo19 was expressed at various levels in multiple tissues of wild-type mice but could not be detected in cross-striated (skeletal, heart) muscles and brain (Figure 1E and Supplementary Figure S1C). The highest levels of Myo19 were detected in the kidney and liver, while lower levels were recorded in the intestine, skin and lungs (Figure 1E). In organs associated with the immune system, e.g., spleen, lymph nodes, thymus and isolated peritoneal macrophages, Myo19 was also detectable. Interestingly, the spleen from adult female Myo19^−/−^ mice was significantly enlarged and slightly, but not significantly, from adult males (Figure 1G,H). Of note in this regard, no alterations in the weight of the thymus and lymph nodes were observed (Supplementary Figure S1D,E). Examination of kidneys from Myo19^−/−^ mice showed no obvious differences in weight and histological characteristics in comparison with kidneys from wild-type mice (Figure 1I,J).

### 3.2. Generation and Characterization of Myo19-Deficient Mouse Embryonic Fibroblasts

**Figure 2:**
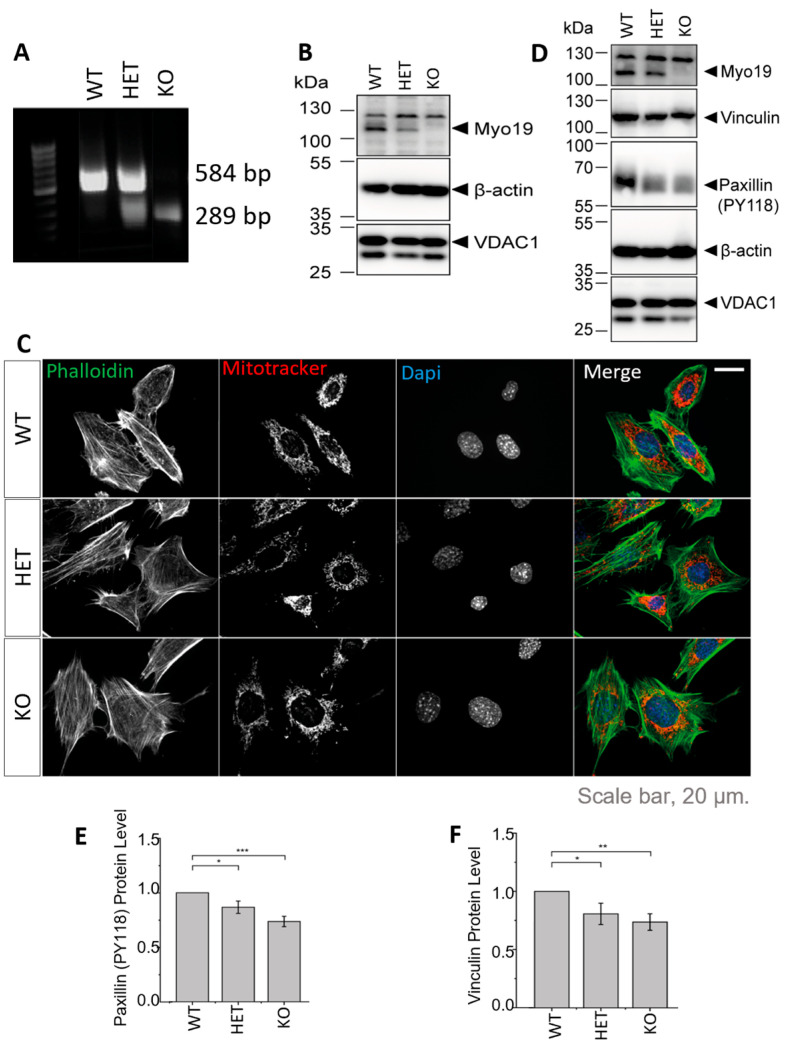
Generation and characterization of Myo19-deficient mouse embryonic fibroblasts (MEFs). (**A**) PCR genotyping of embryos from which the MEFs were derived. (**B**) Immunoblot of MEFs from Myo19 (WT), (HET) and (KO) cell clones. (**C**) Representative images of indicated MEF cell clones stained with MitoTracker Orange, FITC-Phalloidin and DAPI. Scale bar, 20 µm. (**D**) Immunoblot of MEF cells probed with antibodies as indicated. (**E**,**F**) Quantification of paxillin pY118 (**E**) and vinculin (**F**) protein levels normalized to β-actin and in comparison with WT. Data are represented as mean ± SEM and are from 5 independent experiments. *** *p* ≤ 0.001; ** *p* ≤ 0.01; * *p* ≤0.05 (Mann–Whitney U-test).

To study the specific role of Myo19 in mitochondrial dynamics, we generated immortalized mouse embryonic fibroblasts (MEFs) of all the genotypes that arise from *Myo19*^+/–^ × *Myo19*^+/–^ crosses (Figure 2A). The genotypes correlated with the protein levels of Myo19 (Figure 2B). Fluorescence imaging of mitochondria and actin filaments revealed no morphological differences between genotypes (Figure 2C).

Myo19^−/−^ MEFs detached more readily from the culture surface, similar to earlier observations with Myo19^−/−^ HEK cells [17]. This prompted us to examine the levels of proteins participating in cell adhesion by immunoblotting (Figure 2D). Phosphorylated paxillin levels were reduced in both HET and KO MEFs compared with WT MEFs (Figure 2E). Vinculin levels were also reduced in HET and KO MEFs (Figure 2F). These changes are consistent with reduced cell adhesion.

### 3.3. Lack of Myo19 Disrupts the Cristae of Mitochondria

**Figure 3:**
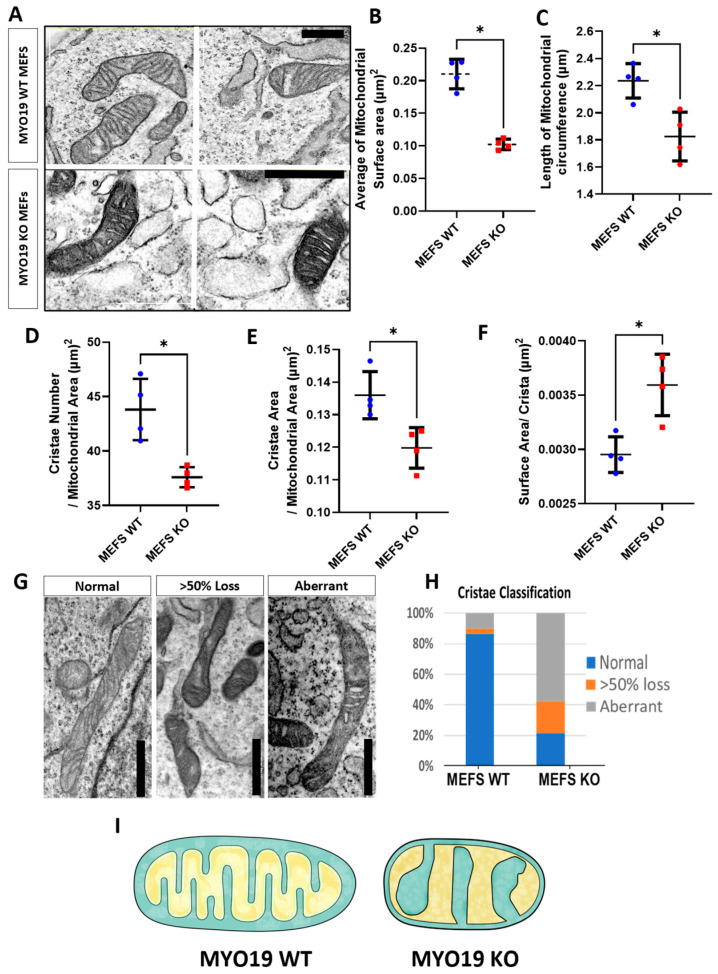
Myo19 loss disturbs cristae organization and structure. (**A**) Representative TEM images of mitochondrial ultrastructure in Myo19 WT and MYO19 KO immortalized MEFs. Scale bar, 0.5 µm. (**B**,**C**) Quantification of mitochondrial surface area (**B**) and circumference (**C**). (**D**–**F**) Quantification of cristae number per mitochondrial area (**D**), cristae surface area per mitochondrial area (**E**) and surface area per crista (F), respectively. Data are shown as mean ± SD of replicate means. Each dot represents one independent biological replicate (n = 4 per group). Total number of analyzed mitochondria: n = 160 WT and n = 180 KO mitochondria. * *p* ≤ 0.05 (Mann–Whitney U-test). (**G**) Representative images of different cristae morphologies. (**H**) Quantification of different cristae morphologies. (**I**) Scheme representing the difference in mitochondrial morphology between WT and KO MEFs.

To further explore the effects of depleting Myo19 on mitochondrial morphology and structure, an ultrastructural analysis of MEFs using TEM was carried out under blind conditions to rule out any bias. In Myo19 WT MEFs, the mitochondria showed a normal homogeneous cristae distribution throughout the mitochondrial segments (Figure 3A). In contrast, Myo19^−/−^ MEFs contained mitochondria with altered cristae organization and darker matrices (Figure 3A). Importantly, Myo19^−/−^ mitochondria had a reduced surface area and circumference compared with WT mitochondria (Figure 3B,C).

To quantify the cristae phenotype, cristae organization was classified into three different categories: (i) normal, (ii) >50% loss and (iii) aberrant (Figure 3G,H). Normal cristae are defined as well-organized, parallel inner membrane protrusions. The other two phenotypes have a loss of cristae projections for more than 50% of the mitochondrial surface area or a complete disruption and distortion in the cristae morphology. The aberrant phenotype is characterized by a swollen and irregular cristae shape with wide cristae junctions. In Myo19 WT cells, mitochondria cristae organization was mainly normal and only a small proportion of mitochondria showed >50% loss or aberrant cristae. In contrast, mitochondria in Myo19^−/−^ cells had, in the majority, aberrant cristae and the residual ones in equal parts of >50% loss and normal cristae (Figure 3H). Moreover, Myo19^−/−^ mitochondria had fewer cristae, reduced total cristae area and increased surface area per crista (Figure 3D,F). A schematic rendering depicts the differences in inner mitochondrial membrane organization that were observed between WT and Myo19^−/−^ MEFs (Figure 3I).

**Figure 4:**
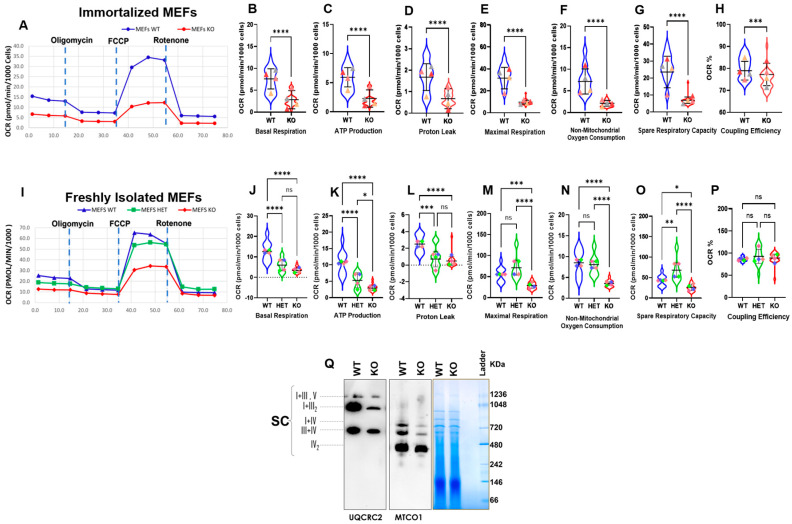
Impaired oxygen consumption rates and supercomplex formation in Myo19-KO MEFs. (**A**) Oxygen consumption rates of immortalized WT and Myo19 KO MEFs following sequential drug injections as indicated of oligomycin, FCCP and rotenone. (**B**–**H**) Quantification of basal respiration, ATP production, proton leak, maximal respiration, non-mitochondrial oxygen consumption, spare respiratory capacity and coupling efficiency for immortalized wild-type (blue) and KO (red) MEFs. Data are means with ± SD from 4 independent experiments, each with ≥5 wells per cell line. Statistical analysis: Mann–Whitney U-test; **** *p* ≤ 0.0001; *** *p* ≤ 0.001; ** *p* ≤ 0.01; * *p* ≤ 0.05; ns, not significant. Triangles in different colors indicate means of individual biological replicates for each independent experiment. (**I**) Oxygen consumption rates of freshly harvested WT, HET and KO MEFs following the same treatments as described in (**A**). (**J**–**P**) Quantification of the same parameters for freshly harvested MEFs. Green: heterozygous MEFs. Data are means with ± SD from 4 independent experiments, each with ≥5 wells per cell line. Statistical analysis: one-way ANOVA with Bonferroni post hoc test.; **** *p* ≤ 0.0001; *** *p* ≤ 0.001; ** *p* ≤ 0.01; * *p* ≤ 0.05; ns, not significant. Squares in different colors indicate means of individual biological replicates for each independent experiment. (**Q**) Blue native PAGE of purified mitochondria (100 µg protein) from WT and KO MEFs, probed for complex III (UQCRC2) and complex IV (MTCO1). I = complex I; III = complex III; III_2_ = complex III dimer; IV = complex IV; IV_2_ = complex IV dimer; V = complex V; SC = supercomplexes; N = 2.

Further, to assess whether the alterations in mitochondrial morphology in Myo19^−/−^ MEFs are associated with changes in mitochondrial activity, the Mito Stress Test from Seahorse XF technology was used to measure the oxygen consumption rate (OCR). The measurement included the inhibition of selected OXPHOS steps using different drug treatments. The addition of oligomycin inhibits ATP-linked respiration by blocking ATP synthase. The remaining basal respiration can be attributed to proton leakage. Furthermore, addition of FCCP (carbonyl cyanide-4-(trifluoromethoxy)phenylhydrazone) ionophore leads to an inner membrane potential collapse and a rapid oxygen consumption to maximum levels. The addition of rotenone allows the contribution of OXPHOS to total respiration to be assessed, as it inhibits mitochondrial complex I. The difference between maximal and basal respiration indicates the spare capacity of mitochondrial OXPHOS and can be taken as a marker of cell fitness and adaptability. The remaining oxygen consumption is of non-mitochondrial origin. The proportion of the ATP-linked respiration to the basal respiration describes the coupling efficiency. The initial experiments used immortalized MEFs. Myo19-KO MEFs showed reduced mitochondrial activity compared with WT (Figure 4A). Additionally, they showed reduced basal respiration, ATP production and proton leak and maximal respiration, with modest reductions in spare capacity and coupling efficiency (Figure 4B–H).

To rule out any effect of immortalization on the mitochondrial activity, the test was repeated with freshly harvested MEFs (WT, Het and KO) (Figure 4I). But essentially comparable reductions in mitochondrial activities were noticed in freshly isolated Myo19^−/−^ MEFs (Figure 4J–P). Efficient respiration depends on the formation of respiratory chain supercomplexes (SCs). Therefore, we assessed the abundance of SCs by blue native–PAGE (BN-PAGE) and found that the abundance of SCs was reduced in Myo19^−/−^ MEFs in comparison with wild-type MEFs (Figure 4Q). Taken together, these findings showed reduced mitochondrial respiration for Myo19-KO MEFs.

### 3.4. Ultrastructural Alterations in Mitochondria in Response to the Loss of Motor Receptor Proteins Miro1 and Miro2 and Microtubule-Based Motors Adaptor TRAK1

**Figure 5:**
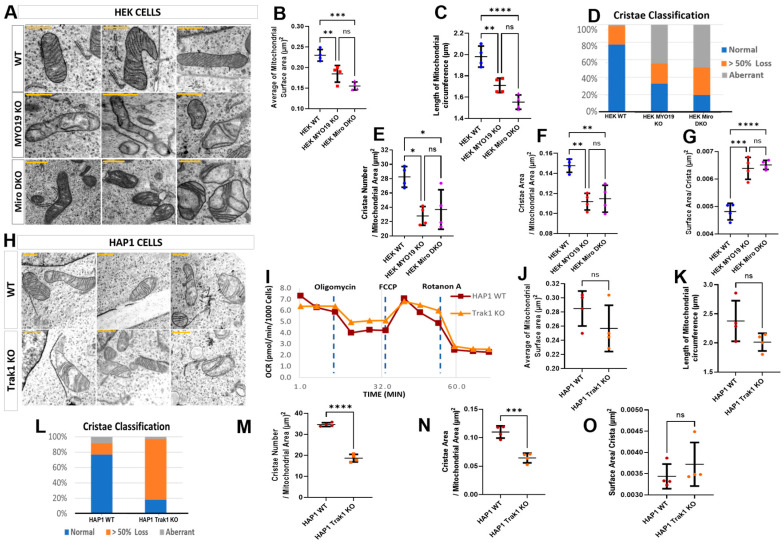
Alterations in mitochondrial ultrastructure due to the lack of Myo19, Miro1/2 and TRAK1. (**A**) Representative TEM images of mitochondria from WT, Myo19-KO and Miro DKO HEK cells (Scale bars = 0.5 μm). (**B**,**C**) Quantification of mitochondrial surface area (**B**) and circumference (**C**). Compared with WT, surface area was reduced by ~20% in Myo19-KO (*p* = 0.0190) and ~32% in Miro DKO cells (*p* = 5.86 × 10^−5^). Circumference was reduced by ~12% in Myo19-KO (*p* = 0.04947) and ~22% in Miro DKO cells (*p* = 4.22 × 10^−4^) compared with WT. (**D**) Quantification of mitochondria according to the classification of their cristae morphology in indicated HEK cells. Classifications: normal; >50% cristae loss; and aberrant cristae. (**E**–**G**) Quantification of cristae number per mitochondrial area (**E**), total cristae surface area per mitochondrial area (**F**), and surface area per crista (**G**) in the indicated HEK cells. Cristae number was reduced in Myo19-KO (*p* = 3.88 × 10^−4^) and Miro DKO (*p* = 0.00337) vs. WT. Cristae surface area was reduced in Myo19-KO (*p* = 4.13 × 10^−5^) and Miro DKO (*p* = 2.95 × 10^−4^) vs. WT. Surface area per crista was increased in Myo19-KO (*p* = 0.00783) and Miro DKO (*p* = 0.00386) vs. WT. Data are shown as mean ± SD of replicate means. Each data point represents one independent biological replicate (n = 4 per group), from 4 different independent blind experiments. Total number of analyzed mitochondria: n = 111 for WT, n = 150 for Myo19-KO, n = 119 for Miro DKO. Statistical analysis: one-way ANOVA with Bonferroni post hoc test. (**H**) Representative TEM images of mitochondria from WT and TRAK1-KO HAP1 cells (Scale bars = 0.5 μm). (**I**) Mitochondrial respiration profiles of WT and TRAK1-KO HAP1 cells following sequential drug injections, measured in two independent experiments. (**J**,**K**) Quantification of mitochondrial surface area (**J**) and circumference (**K**) in HAP1 cells. (**L**) Quantification of mitochondria with different cristae morphologies in WT and TRAK1 KO HAP1 cells. Mitochondria number n = 86 for WT and n = 71 for TRAK1 KO HAP1 cells. (**M**–**O**) Quantification of cristae number per mitochondrial area (**M**), total cristae surface area per mitochondrial area (**N**), and surface area per crista (**O**) in HAP1 cells. Data are shown as mean ± SD of replicate means. Each data point represents one independent biological replicate (n = 4 per group), from 4 different independent blind experiments. Total number of analyzed mitochondria: n = 86 for WT HAP1, n = 71 for TRAK1-KO. Statistical analysis: Mann–Whitney U-test. **** *p* ≤ 0.0001; *** *p* ≤ 0.001; ** *p* ≤ 0.01; * *p* ≤ 0.05; ns, not significant.

To assess whether the loss of MYO19 causes mitochondrial ultrastructural defects comparable with those reported for the absence of its mitochondrial receptor proteins Miro1/2, we compared the mitochondria of Myo19-KO and Miro1/2 double knockout (Miro DKO) HEK cells with each other [17] (Supplementary Figure S2). In both cases, mitochondria showed severely distorted cristae, with either a dramatic reduction in cristae number creating large void spaces or aberrant cristae morphology, similar to that seen in Myo19-KO MEFs (Figure 5A). Mitochondria were smaller in both genotypes, with reduced surface area and circumference compared with WT (Figure 5B,C).

Classification of cristae morphologies revealed comparable alterations for Miro DKO and Myo19-KO mitochondria (Figure 5D). WT mitochondria had a mostly normal cristae organization, with few showing substantial cristae loss or aberrant morphology. However, in both mutants, about half of the mitochondria showed aberrant cristae and an increased level of mitochondria had substantial cristae loss.

Analysis of cristae organization revealed reductions in cristae number and total cristae surface area in both Myo19-KO and Miro DKO mitochondria as compared with WT (Figure 5E,F). But no differences were noted between the mitochondria of the two KO cell lines. In addition, the surface area per crista was comparably increased in both mutants (Figure 5G). Together, these findings showed that MYO19 loss produces structural defects in cristae organization closely matching those caused by Miro1/2 loss, supporting the conclusion that loss of Myo19 is the primary driver of the ultrastructural alterations observed in Miro DKO mitochondria.

TRAK1/2 serve as adaptors between microtubule-based motors and Miro1/2. To analyze the impact of microtubule-based force production on the ultrastructure of mitochondria, the mitochondrial ultrastructure was investigated in commercially available TRAK1 KO HAP1 cells. But unlike mitochondria in Myo19 KO and Miro DKO cells, it was difficult to distinguish mitochondria from WT and TRAK1 KO cells. The size of the mitochondria was comparable between WT and TRAK1 KO cells (Figure 5H). Surface area and circumference did not differ significantly (Figure 5J,K). WT cells were mostly normal, whereas TRAK1 KO mitochondria were dominated by the >50% cristae loss category (Figure 5L). This was also evident in the reduced number of cristae per mitochondrial area in TRAK1 KO mitochondria (Figure 5M) and in the reduced total cristae surface area (Figure 5N). Of note, the surface area per crista was unchanged (Figure 5O).

We were curious to check to what extent this loss of cristae number would affect the respiratory profile of the mitochondria; hence, the Mito Stress Test was performed using Seahorse XF technology to measure the OCR. No significant differences were noted in the OCR consumption between the WT and TRAK1 KO HAP1 cells (Figure 5I). To sum up, both microtubule-based motors and the actin-based motor Myo19 regulate the fine structure of mitochondria through the interaction with Miro1/2. However, the loss of Myo19 mirrors for the most part the loss of Miro1/2.

### 3.5. Myo19 Regulates ER–Mitochondria Contacts

**Figure 6:**
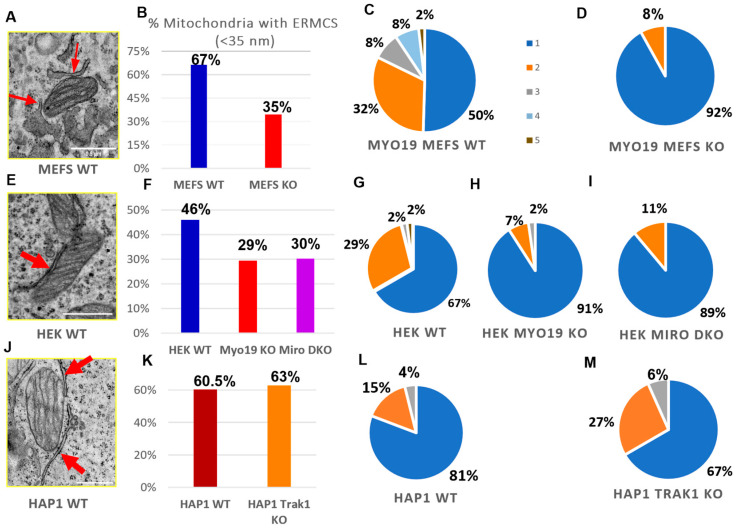
Myo19 and Miro regulate ER–mitochondrial contacts. (**A**) Representative TEM image of ER–mitochondria contacts in WT MEFs. Red arrows point to the ER/mitochondria close contacts (<35 nm). (**B**) Percentage of mitochondria with ERMCS defined by a distance of less than 35 nm between OMM and ER membranes for WT and Myo19 KO MEFS. (**C**,**D**) Number of ER–mitochondria contact sites per mitochondrion between WT (**C**) and Myo19 KO (**D**) MEFs excluding the mitochondria that lack ERMCSs. Color legend indicates number of contact sites per mitochondria. (**E**) Representative TEM image of ER–mitochondria contacts in HEK cells. (**F**) Percentage of mitochondria with ER–mitochondria contact sites of less than 35 nm in WT, Myo19 KO and Miro DKO HEK cells. (**G**–**I**) Number of ER–mitochondria contact sites per mitochondrion in HEK WT (**G**), Myo19 KO (**H**) and Miro DKO cells (**I**). (**J**) Representative TEM image of ER–mitochondria contacts in HAP1 WT cells. (**K**) Percentage of mitochondria with ER–mitochondria contact sites of less than 35 nm in HAP1 WT and TRAK1 KO cells. (**L**,**M**) Number of ER–mitochondria contact sites per mitochondrion in HAP1 WT (**L**) and TRAK1 KO cells (**M**).

To check whether Myo19 plays a role in the contacts between the ER and the mitochondria, TEM was used to quantify endoplasmic reticulum–mitochondria contacts (ERMCs) per section, defined by an ER–mitochondria membrane distance smaller than/equal to 35 nm. Remarkably, mitochondria with one or more ERMCSs were abundant in WT MEFs but reduced by 32% in Myo19-KO MEFs (Figure 6A,B). The number of ERMCs per mitochondrion was also reduced. While WT MEFs showed a distribution ranging from 1 to 5 contacts (Figure 6C), Myo19-KO MEFs had mostly just a single contact and, rarely, two (Figure 6D).

We further examined the abundance of ERMCSs in Myo19 KO HEK cells, Miro DKO HEK cells and TRAK1 KO HAP1 cells. In Myo19 KO and Miro DKO HEK cells, the percentage of mitochondria with no ERMCSs increased in comparison with WT HEK cells. Myo19-KO and Miro DKO HEK cells showed a 17% and 16% increase in the number of mitochondria without any ERMCSs, respectively (Figure 6F). In addition, the number of ERMCSs per mitochondrion was altered. WT HEK cells had 1–3 or 5 contacts, while most Myo19-KO (91%) and Miro DKO (89%) mitochondria had one contact and the remaining had two (Figure 6G–I).

Interestingly, the number of mitochondria with ERMCSs did not differ between WT and TRAK1 KO HAP1 cells (Figure 6J,K). Furthermore, in those mitochondria with ERMCSs, the frequency of ERMCSs was also comparable (Figure 6L,M), indicating that TRAK1 does not regulate ER–mitochondria contacts. These findings support a role for Miro in regulating ER–mitochondria communication via Myo19.

## 4. Discussion

Mitochondrial structure and dynamics are closely linked to function, and alterations in either can contribute to neurological, metabolic or cardiovascular disorders Here, we characterize the role of the mitochondria-associated actin-based motor protein Myo19 by generating and analyzing Myo19-deficient mice and cells. Lack of Myo19 led to alterations in cristae organization and reduced numbers of ERMCSs. These structural alterations were accompanied by an impaired OCR. Comparable results were obtained in the absence of the mitochondrial Myo19 receptors Miro1/2 (Miro DKO) but not in the absence of the microtubule motor adaptor TRAK1 [35,36,37,38,39].

Although the mitochondria in Myo19-deficient cells showed a clearly altered IMM architecture and a reduced number of ER contacts, the mice exhibited no other apparent phenotype. The offspring genotype distribution matched the expected Mendelian ratio. The Myo19-deficient mice had a comparable body weight and lifespan to their wild-type siblings. Mutations on chromosome 11 in mice in the area of the Myo19 gene have been associated with prenatal and postnatal lethality and male and female infertility [40]. However, the present results make it unlikely that these mice contained mutations directly in Myo19.

In adult mice, the Myo19 protein expression was high in the kidney and liver and lower in the intestine, skin and lungs. Myo19 was also detectable in organs that play an important role in the immune system, such as the spleen, lymph nodes or thymus. Surprisingly, Myo19 protein could not be detected in brain, heart and skeletal muscle tissues, although databases indicate that Myo19 is at least transcribed in these organs [41].

Interestingly, in adult Myo19-deficient mice, the spleen was significantly enlarged in females and slightly, but not significantly, in males. However, histological examinations revealed no obvious abnormalities in the main constituents of the spleen, the white and the red pulp. Mitochondria are well known to have a great impact on T cell fate and function [42]. But it remains to be seen which cells contribute to splenomegaly in the Myo19-deficient female mice. Splenomegaly has also been observed in mice that lacked mitochondrial complex III in Treg cells [43]. In Myo19 KO MEFs, the formation of supercomplexes (SCs) containing complexes 3 and 4 was reduced. Additionally, mitochondrial dynamics that are affected by the lack of Myo19 have been shown to impact immunological signaling [44]. Therefore, alterations in mitochondrial dynamics in Myo19-deficient mice may explain the observed splenomegaly.

A recent genome-wide association study of clinically diagnosed chronic kidney disease (CDK) identified Myo19 as a likely candidate gene relevant for kidney function [45]. Oxidative stress from mitochondrial dysfunction has been identified as a cause of kidney diseases [46]. In fact, increased levels of reactive oxygen species (ROS) have been reported in Myo19-deficient cells [17]. However, no histological phenotype in kidneys of Myo19-deficient mice could be detected.

In addition, mutations in Myo19 have been linked with different cancers. Paired-end RNA-seq studies associated Myo19 fusions with breast cancer [47]. Two missense mutations in the Myo19 gene were identified in persons with familial glioma, a cancer that accounts for most of malignant primary brain tumors [48]. We noticed that Myo19-deficient MEFs did not attach as well to the substrate as wild-type MEFs. The analysis of focal adhesion proteins in Myo19-deficient MEFs showed a significant reduction in vinculin and pY118 paxillin levels. Moreover, immunofluorescence labeling of focal adhesion complexes revealed that cells lacking Myo19 have less prominent focal contacts at the cell periphery. These findings are in agreement with previous results obtained in HEK cells [17]. The focal adhesion phenotype could be rescued in these cells by quenching reactive oxygen species, suggesting that elevated levels of ROS, due to the loss of Myo19, modify focal adhesions [17]. These alterations in focal adhesions might contribute to cancer development.

We envisioned that the reported stochastic failure in cytokinesis and the asymmetric distribution of mitochondria to daughter cells in cultured Myo19-deficient cells [17,23] would lead to developmental defects. However, no obvious developmental defects were noticed in Myo19-KO mice indicating a remarkable plasticity during development and the presence of compensatory mechanisms. This is even more surprising, because we noticed, in all cells analyzed, alterations in cristae architecture and reduced numbers of mitochondrial contacts with the ER. The altered cristae organization was accompanied by a reduction in supercomplexes and an impaired respiration. The observed reduction in supercomplex formation was not due to altered levels of individual OXPHOS complexes I–V, as we have reported previously that their levels were not altered [17].

A reduction in cristae count and a widening of cristae junctions upon loss of Myo19 has recently also been reported by Shi et al. [24]. Additionally, a reduction and structural changes in cristae as well as a reduced number of ER contacts were reported for mitochondria in Miro1/2-double-deficient cells [25]. We report here that the alterations in mitochondria morphology and ER contacts were largely identical for Myo19 KO and Miro DKO cells. We noted that cristae number was also reduced in adaptor TRAK1-deficient cells in agreement with work by Lee et al. [49]. They reported that downregulation of TRAK1 or the expression of truncated TRAK1 (hyrtTRAK1) affected mitochondria size and induced disorganized and disrupted cristae structures. The consequences of a loss of TRAK2 or a combined loss of TRAK1 and TRAK2 have not been determined yet. However, based on the comparable morphological alterations in mitochondria lacking either Myo19 or Miro1/2, Myo19 appears to be largely responsible for the observed alterations and to be positioned upstream of Miro1/2.

Hackenbrock [50,51,52] discovered that mitochondria can adopt either of two states, which he termed condensed and orthodox. The condensed state is characterized by contracted mitochondria, wide cristae and a very dense matrix. The orthodox state is characterized by expanded mitochondria, compacted cristae and a less-dense matrix. The two states showed a direct correlation with energy levels. The dynamic change in cristae shape is known as “cristae remodeling” [53]. Particularly, Myo19-deficient MEFs showed concomitantly with cristae remodeling a denser matrix in comparison with WT MEFs, as is also characteristic of the condensed state. This observation further indicates that Myo19 regulates, in conjunction with the structural transformations, the functional states of mitochondria.

It has been shown that ERMCs are contributing to mitochondrial fission [54,55]. Since the loss of Myo19 is causing a reduction in ERMCs, it was rather surprising to find that mitochondria were smaller in Myo19-deficient cells. Indeed, a recent report showed that knockdown of Myo19 resulted in elongated mitochondria [26]. However, evidence is mounting that actin filaments also contribute to mitochondrial fusion [56]. In previous work, we showed that in HEK interphase cells the lack of Myo19 did not affect mitochondrial fusion but failed to inhibit fusion at prometaphase [17]. These opposing observations may be explained by the functions of actin in both fission and fusion [56].

How could the OMM proteins Myo19 and Miro control the organization of the IMM? We would like to offer two mutually non-exclusive possibilities. Miro and Myo19 have been suggested to be linked to the MICOS complex and thereby transduce force exerted by Myo19 on the outer membrane to the inner membrane. Immunoprecipitation of Myo19 and Miro led to the co-precipitation of the MICOS components [24,25,57]. Furthermore, Myo19 has been suggested to interact with metaxins that are believed to be part of the SAM50 complex, which in turn interacts with the MICOS complex [21,24,26,58]. A lack of force transduction on the MICOS complex and the IMM in Myo19- or Miro-deficient cells might alter IMM architecture as previously suggested by Shi et al. [24] for Myo19. Alternatively, Myo19 and Miro could regulate IMM organization by being part of the ERMCS. Loss of Myo19 and Miro reduces the number of ERMCSs. Several proteomics studies classified Myo19 and Miro as ERMCS proteins [59,60]. ERMCSs facilitate lipid transfer between the two organelles and lipid composition of the IMM is known to influence its organization [6,61,62,63,64]. Interestingly, the MICOS also contributes to the transfer of lipids to the IMM [65,66]. Therefore, Myo19 might influence lipid transfer to the IMM [57] and hence structure, by being linked to both the MICOS complex and the ERMCSs. Whether any of the two possibilities explain the cristae phenotype observed here in Myo19- and Miro-deficient cells, remains to be investigated further.

## Data Availability

The original contributions presented in this study are included in the article/Supplementary Materials. Further inquiries can be directed at the corresponding author.

## Supplementary Materials

The following supporting information can be downloaded at: https://www.mdpi.com/article/10.3390/cells14211657/s1, Figure S1: Myo19 knockout mice exhibited no discernible variations across various tissues. (A) Quantification of testis weight of wild-type (WT), heterozygous (HET) and homozygous (KO) Myo19 mice. n ≥ 3, error bars represent ±SEM. (B) Histological sections of mouse testes from WT and Myo19 KO animals as indicated. Paraffin embedded testis sections were stained with Hematoxylin and Eosin (H&E). (C) Tissue expression of Myo19 protein. Immunoblot analysis of tissue homogenates from WT and Myo19 KO mice were probed with the indicated antibodies. Tissues are indicated at the top of the panels. Several separate blots were assembled as marked by dashed lines. (D,E) The weight of isolated thymus (D) and lymph nodes (E) of adult female and male mice of the indicated genotypes was determined. n ≥ 6, error bars represent ±SEM; Figure S2: Characterization of Miro double knockout HEK cells by immunoblotting with the indicated antibodies.
