# Supplementary material for: Myosin-19 and Miro Regulate Mitochondria–Endoplasmic Reticulum Contacts and Mitochondria Inner Membrane Architecture
Source: Cells. 2025 Oct 23;14(21):1657. doi: 10.3390/cells14211657 (PMC12610653; doi:10.3390/cells14211657)
Supplement: Supplementary file 1 [file cells-14-01657-s001.zip › cells-3774368-supplementary.pdf]

Supplementary Information

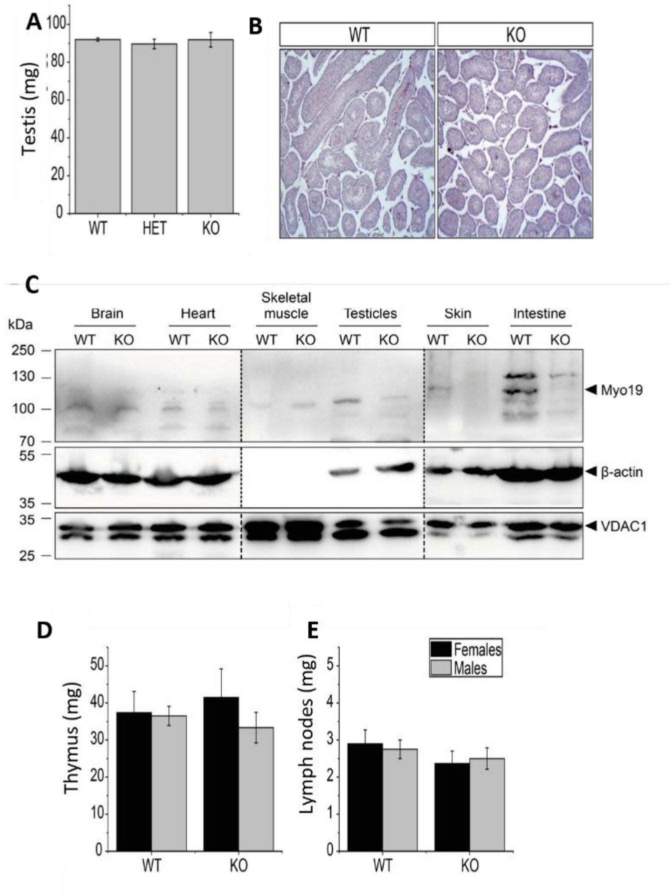

Supplementary Figure S1. Myo19 knockout mice exhibited no discernible variations across various tissues. (A) Quantification of testis weight of wild-type (WT), heterozygous (HET) and homozygous (KO) Myo19 mice. n≥3, error bars represent ±SEM. (B) Histological sections of mouse testes from WT and Myo19 KO animals as indicated. Paraffin embedded testis sections were stained with Hematoxylin and Eosin (H&E). (C) Tissue expression of Myo19 protein. Immunoblot analysis of tissue homogenates from WT and Myo19 KO mice were probed with the indicated antibodies. Tissues are indicated at the top of the panels. Several separate blots were assembled as marked by dashed lines. (D,E) The weight of isolated thymus (D) and lymph nodes (E) of adult female and male mice of the indicated genotypes was determined. n≥6, error bars represent ± SEM.

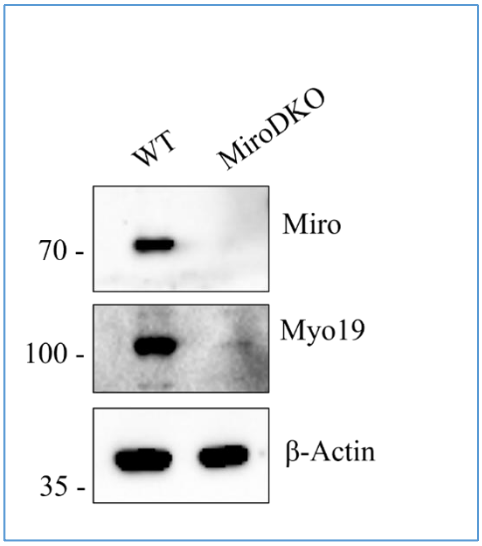

Supplementary Figure S2. Characterization of Miro double knockout HEK cells by immunoblotting with the indicated antibodies.
